# Structural Requirements and Plasticity of Receptor-Binding Domain in Human Coronavirus Spike

**DOI:** 10.3389/fmolb.2022.930931

**Published:** 2022-07-12

**Authors:** Yajuan Li, Peiyi Zheng, Tingting Liu, Cuixiao Shi, Bo Wang, Yuanhong Xu, Tengchuan Jin

**Affiliations:** ^1^ Department of Clinical Laboratory, The First Affiliated Hospital of Anhui Medical University, Hefei, China; ^2^ Laboratory of Structural Immunology, CAS Key Laboratory of Innate Immunity and Chronic Disease, School of Basic Medical Sciences, Division of Life Sciences and Medicine, University of Science and Technology of China, Hefei, China

**Keywords:** human coronavirus, receptor-binding domain, structure, plasticity, mutation

## Abstract

The most recent human coronaviruses including severe acute respiratory syndrome coronavirus-2 causing severe respiratory tract infection and high pathogenicity bring significant global public health concerns. Infections are initiated by recognizing host cell receptors by coronavirus spike protein S1 subunit, and then S2 mediates membrane fusion. However, human coronavirus spikes undergo frequent mutation, which may result in diverse pathogenesis and infectivity. In this review, we summarize some of these recent structural and mutational characteristics of RBD of human coronavirus spike protein and their interaction with specific human cell receptors and analyze the structural requirements and plasticity of RBD. Stability of spike protein, affinity toward receptor, virus fitness, and infectivity are the factors controlling the viral tropisms. Thus, understanding the molecular details of RBDs and their mutations is critical in deciphering virus evolution. Structural information of spike and receptors of human coronaviruses not only reveals the molecular mechanism of host–microbe interaction and pathogenesis but also helps develop effective drug to control these infectious pathogens and cope with the future emerging coronavirus outbreaks.

## Introduction

The zoonotic coronaviruses (CoVs) undergo significant mutation and genome recombination during evolution to adapt to new hosts and facilitate cross-species transmission to the ultimate human host. These coronaviruses may cause central nervous system, respiratory and gastrointestinal diseases in humans and animals (Gupta et al., 2020; Hartenian et al., 2020; Pellegrini et al., 2020), causing serious damage to human health and the global economy. As the largest and enveloped viruses, RNA genomes of CoVs with positive single strand range from 27 to 32 kb (Lai et al., 2007; Lu and Liu 2012). According to the criteria of serological response and genome sequence homology, coronaviruses are divided into three different groups: mammalian groups I and II, and avian group III (Holms and Lai, 1996). Based on antigenic characters, the viruses can be classified into four genera: *Alpha-*coronavirus, *Beta-*coronavirus, *Gamma-*coronavirus, and *Delta-*coronavirus (Woo et al., 2009).Up to now, about 16 different coronavirus strains have been identified, among which 7 coronaviruses infect human beings (hCoVs) (Table 1). HCoV-229E and hCoV-NL63 belong to *Alpha-*CoVs, while HCoV-OC43, HKU1, Middle East respiratory syndrome CoV (MERS-CoV) (Wevers and van der Hoek, 2009; Lu et al., 2015), severe acute respiratory syndrome CoV-1 (SARS-CoV-1), and severe acute respiratory syndrome CoV-2 (SARS-CoV-2) (Lu et al., 2020) belong to lineage A, A, C, B, and B of *Beta-*CoVs, respectively. Some hCoVs, such as 229E, NL63, OC43, and HKU1, cause mild common cold-like and endemic respiratory symptoms, while SARS-CoV-1, MERS, and SARS-CoV-2 can cause moderate to severe human respiratory diseases. It is this human-to-human transmission that is attracting global attention. Since 2002, the SARS-CoV-1 has infected over 8000 people and caused acute respiratory distress syndrome and fatal respiratory failure, with a mortality rate of ∼10%. Since 2012, MERS-CoV has infected 2000 people and displayed ∼36% mortality rate. As of May 2022, SARS-CoV-2 has infected 518 million people and over 6 million deaths have been reported globally (WHO, 2022). Some coronaviruses have circulated for a long time and are difficult or impossible to eliminate due to their adaptability through mutation and recombination. Different variants of SARS-CoV-2 have been identified (e.g. highly transmissible Delta and Omicron variants) with more critical residue mutations, creating a new crisis for the current vaccine strategy.

**TABLE 1:** Classification of human coronaviruses.

Coronaviridae genera	Discovery	Strain	Spike RBD	Cellular receptor	Structure (PDB code)	Hosts reference
Alpha-coronavirus	1966	HCoV-229E	Domain B 308–325; 352–359; 404–408	APN (CD13)	6ATK	Bats, alpacas, and camelids Corman et al. (2015); Corman et al. (2016); Crossley et al. (2012); Huynh et al. (2012)
	2004	HCoV-NL63	Domain B 493–503; 531–541; 585–590	ACE2, heparan sulfate	3KBH	Bats Donaldson et al. (2010); Huynh et al. (2012)
Beta-coronavirus	1967	HCoV-OC43	Domain A	9-*O*-Acetylated sialic acid	6NZK	Rodents Forni et al. (2017); Su et al. (2016)
	2003	SARS-CoV	Domain B 424–494	ACE2	3SCI	Bats and palm civets Hu et al. (2017); Song et al. (2005); Wang et al. (2005)
	2005	HCoV-HKU1	Domain A	9-*O*-Acetylated sialic acid	5I08	Rodents Forni et al. (2017); Su et al. (2016)
	2012	MERS-CoV	Domain B 424–454 and Domain A	DPP4 and sialic acid	4L72	Bats and camels Alagaili et al. (2014); Azhar et al. (2014); Cui et al. (2019); Hu et al. (2015)
	2019	SARS-CoV-2	Domain B 438–498	ACE2	6LZG	Bats and pangolins Lam et al. (2020); Zhou et al. (2020)

**FIGURE 1:**
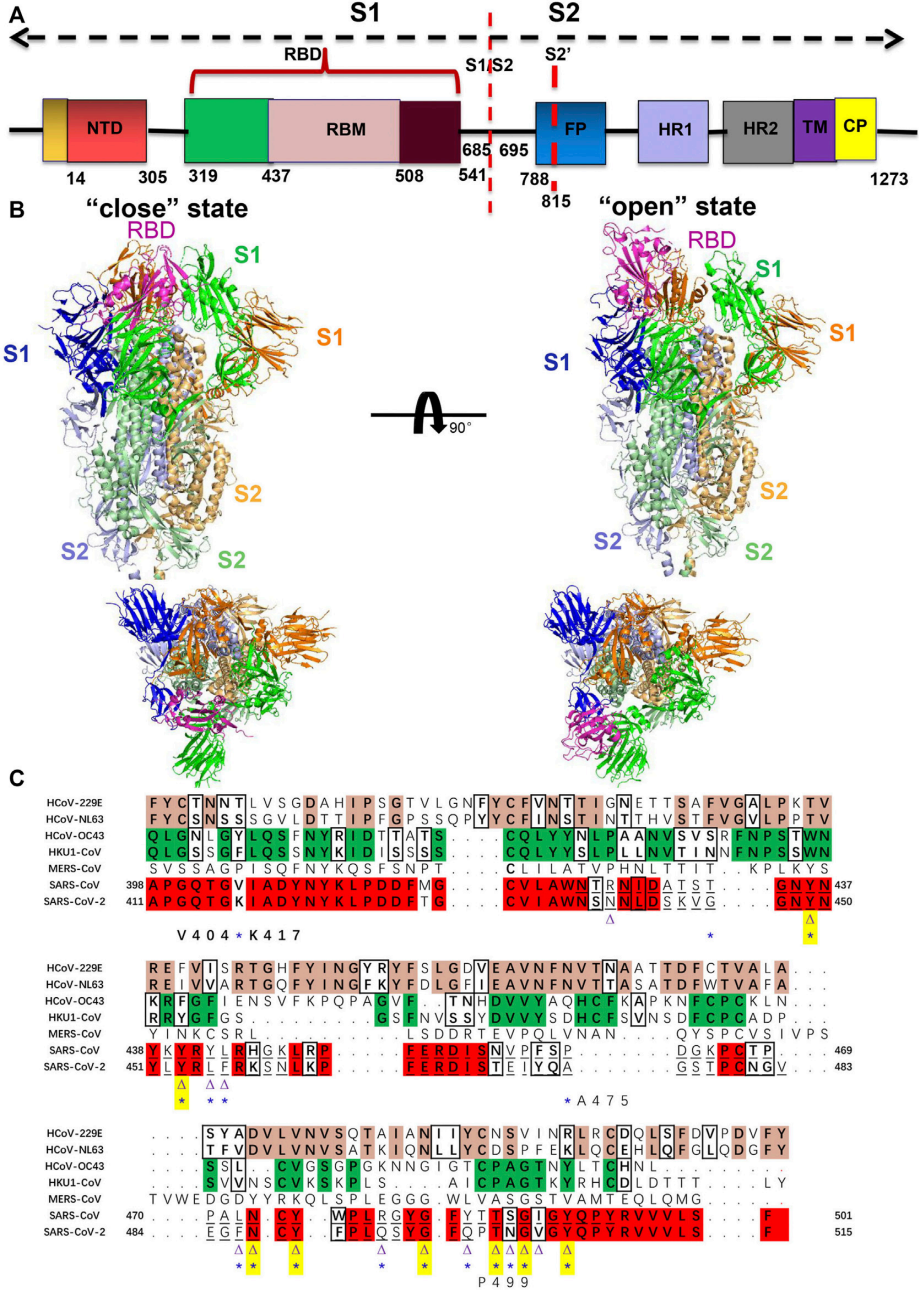
**(A)** Structure of S protein of SARS-CoV-2 colored by the domain. S1/S2: furin cleavage sites; S2’: S2′ protease cleavage site; FP: fusion peptide; HR1: heptad repeat 1; HR2: heptad repeat 2; TM: transmembrane domain; CP: cytoplasmic domain fusion. **(B)** Structure of the human coronavirus spike trimer that comprised S1 and S2 subunits is shown in cartoon representation. Side and top views of the SARS-CoV-2 ectodomain trimer with open (PDB code: 6vyb) and closed (PDB code: 6vxx) conformation (Walls et al., 2020). The three protomers of S1 and S2 are shown in blue, green, and orange, and light blue, pale green, and light orange, respectively. The RBD that possesses different conformations is colored in magenta. **(C)** Sequence analysis and alignment among human coronavirus spike RBD. Sequences alignment of spike RBD from HCoV-229E, NL63, OC43, HKU1, MERS-CoV, SARS-CoV-1, and SARS-CoV-2 are used through ClustalW at https://www.genome.jp/tools-bin/clustalw (Bahar et al., 1997). The conserved residues between 229E and NL63 are highlighted in pink and similar characteristic residues in the black box; the conserved residues between OC43 and HKU1 are highlighted in green and similar characteristic residues in the black box. The conserved residues between SARS-CoV-1 and SARS-CoV-2 are highlighted in red and similar characteristic residues in the black box. Underlined residues represent the RBM region. The ACE2-binding residues of SARS-CoV-1 (16 residues) and SARS-CoV-2 (17 residues) that participate in interaction with ACE2 are indicated by a purple triangle and a blue asterisk, respectively. Out of 16 residues that bind to the ACE2 in SARS-CoV-1, 8 amino acid residues are strictly conserved in SARS-CoV-2, which is indicated by both the triangle and asterisk as well as highlighted in yellow. The substitution of Val404 of SARS-CoV-1 RBD with Lys417 in SARS-CoV-2 RBD is the most prominent mutation that increases affinity to ACE2. P499 and A475 residues in SARS-CoV-2 are unique that involve in the critical interaction with ACE2.

Virus infections are initiated by viruses binding to host cellular receptors. In coronaviruses, the spikes are composed of a large ectodomain, a single-pass transmembrane domain, and a short intracellular domain. As a class I fusion protein, spike glycoprotein is initially synthesized as a single polypeptide, such as SARS-CoV-2 polypeptide with 1300 amino acids (Figure 1A). It is then further processed by host and endolysosomal proteases into an N-terminal S1 subunit and a C-terminal S2 subunit, which are responsible for binding of the virus and host cell receptor and fusion of the viral and cellular membranes, respectively. The receptor-binding domain (RBD) is localized in the C-terminal region of the S1 subunit, with ∼200 amino acids, and consists of a core and external subdomains (Lu et al., 2013; Lu et al., 2015). The host specificity of CoVs is determined by the club-shaped trimeric spike protein located on the envelope (Figure 1B). The spike ectodomain is a key target for diagnosis and treatment for infected individuals. Serological antibody and antigen detection confirm coronaviridae infection and antibody titers facilitate identification of potentially infected individuals (Casadevall and Pirofski 2020). In addition, the S ectodomain, as an important antigen, provokes B cells in the body to produce neutralization antibodies.

Recent outbreaks of SARS-CoV-2 are homologous to other human coronaviruses with ∼80% protein sequence identity (Shereen et al., 2020), but spread more rapidly and have higher infectivity. Further analyzing the receptor-binding motifs (RBMs) in RBD showed that the sequence identity falls to 50%, which indicates higher variability of the binding residues. Figure 1C represents the sequence alignment of multiple hCoV spike RBDs. Therefore, the Spike RBD is a direct druggable target for vaccine design and developing small molecules in the fight against HCoVs. The understanding of the structure and mutational characteristics of spike RBD at the atomic level provides important information and clues about the interactions between coronaviruses and hosts and assists with structure-based drug and vaccine design.

To date, most structures of the human coronavirus spike ectodomain in the pre-fusion and post-fusion have been determined by cryo-EM and X-ray crystallography. These studies reveal their respective typically homotrimeric conformations. In each protomer, it is composed of S1 and S2 subunits. In addition, the analysis and comparison of spike RBD structures between different human coronaviruses displays sequence variations and structural conformation changes that determine host range and viral infectivity.

In this review, we review some of these recent structural and mutational characterizations of RBD of human coronavirus spike protein and their interactions with specific human cell receptors and the mutation hotspots of recent SARS-CoV-2 RBM. Conserved conformation and differences of S RBD protein and interaction with receptor make them various tropisms of specific receptor binding and affinity, and infectivity. Structural information of spike and receptors of human coronaviruses not only reveals the molecular mechanism of host-microbe interaction and pathogenesis, but also helps develop effective drug to control these infectious pathogens, and cope with the future emerging coronavirus outbreaks.

## Overall Structure of Human Coronavirus Spike RBD

**FIGURE 2:**
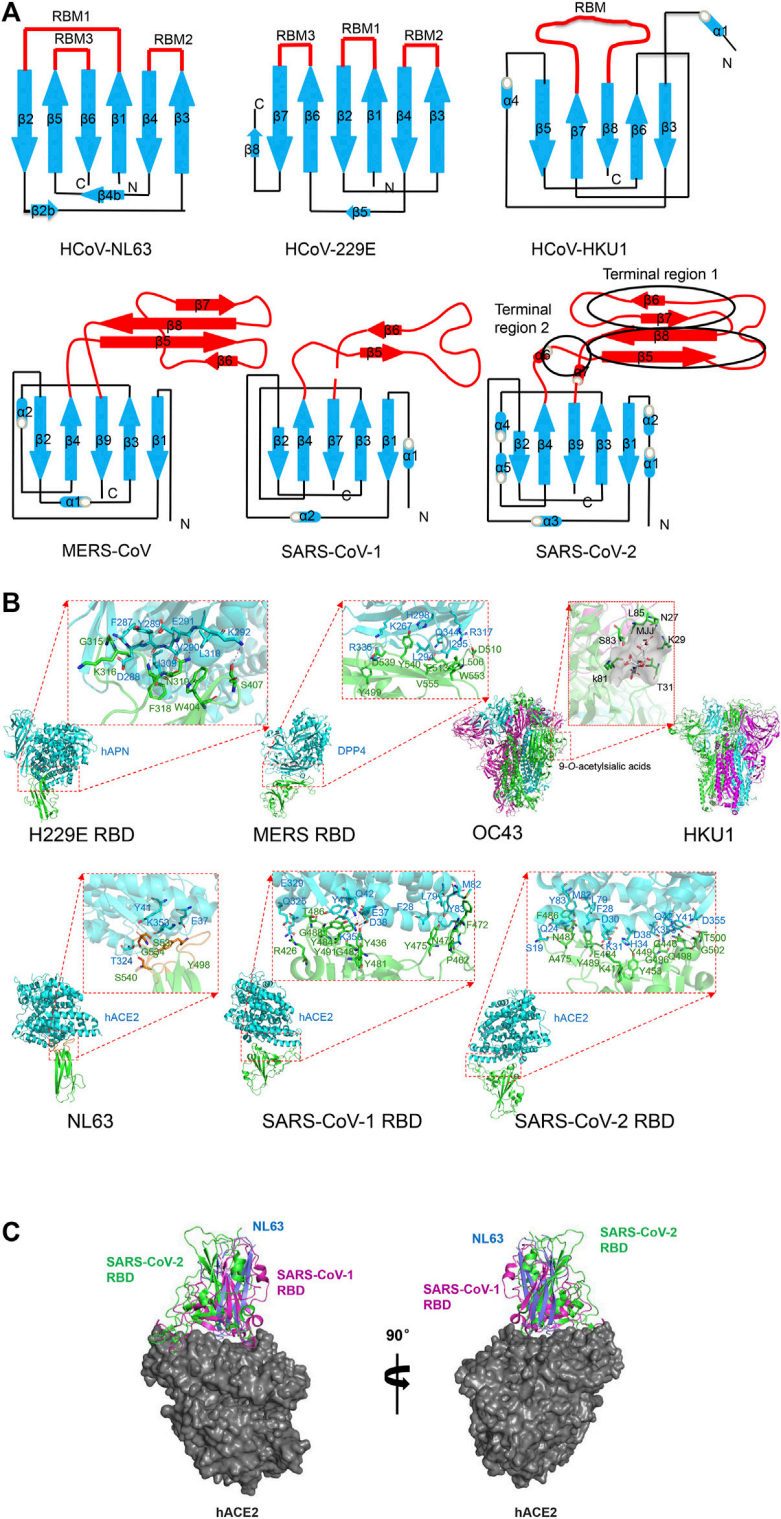
Structure of human coronavirus spike RBD–receptor complexes. **(A)** Schematic of the overall structure of human coronavirus spike RBD monomer. Receptor-binding subdomain and core subdomain are indicated in red and blue color, respectively. **(B)** Cartoon representation of human coronavirus RBD complexes with their primary receptors. 229E with hAPN (PDB code: 6ATK) (Wong et al., 2017). MERS with DPP4 (PDB code: 4L72) (Wang et al., 2013). Residues K267, H298, R336, R317, and Q344 on blade 4 and blade 5 of DPP4 interact with D510, E536, D537, and D539 residues on MERS RBD with hydrogen bonds or salt bridges. A hydrophobic core is formed between Leu294 and Ile295 of DPP4 and Leu506 and V555 of MERS RBD. OC43 with 9-*O*-acetylsialic acids (PDB code: 6NZK) (Tortorici et al., 2019). HKU1 (PDB code: 5I08) (Kirchdoerfer et al., 2016). 9-*O*-Acetylsialic acids and the surface of binding cave of two hydrophobic pockets are colored in gray. NL63 with hACE2 (PDB code: 3KBH) (Wu et al., 2009). Hotspot on ACE2 involving in K353 and corresponding Y498 and S535 on NL63 side chains are shown in red. SARS-CoV-1 with ACE2 (PDB code: 3SCI) (Wu et al., 2012). Various hydrogen bonds and salt bridges were found at the interface between conserved residues including Thr486, Tyr475, Tyr491, and Tyr481 of SARS-CoV-1 RBD and Gln42, Glu37, Tyr83, and Arg353 on ACE2. Residues Phe28, Leu79, and Met82 of hACE2 and Phe472 of SARS RBD form a hydrophobic core. SARS-CoV-2 with hACE2 (PDB code: 6LZG) (Wang Q. et al., 2020).The interaction residues Lys417, Asn501, Gln493, Ser494, Leu452, F486, L455, Tyr489, and Ala475 on RBM and Lys31, Glu35, Asp38, and Lys353 of ACE2 are indicated. Residues Phe28, Leu79, and Met82 of hACE2 and Phe486 of SARS-CoV-2 RBD form a hydrophobic core. In all figures, the RBD and receptors are shown in green and purple, respectively. The interacting residues are shown as sticks. Dashed lines represent hydrogen bonds and salt bridges. **(C)** Structural comparison of epitope of RBDs binding to the same hACE2. The secondary structure elements of RBDs from NL63 (PDB ID: 3KBH), SARS-CoV-1 (PDB ID: 3SCI), and SARS-CoV-2 (PDB ID: 6LZG) are shown in blue, magenta, and green, respectively. The surface of hACE2 is colored in gray. All structural figures were generated using PyMOL software (http://pymol.org).

Human coronaviruses are distributed into two genera: α-and β-CoVs. Their spike proteins form the single-pass membrane trimers on the viral membrane, presenting pre- and post-fusion conformations to further activate coronaviridae cellular entry by respective receptors, with the S1 domain containing RBD and the S2 domain containing fusion peptide and heptad repeats. Figure 2A shows a schematic representation of spike RBD between HCoV. The S1 subunit is divided into domains A, B, C, and D. Domain A and domain B are termed the N-terminal domain (NTD) and C-terminal domain (CTD) of S1, respectively. In β-HCoVs, the canonical core of domain B (also termed as RBD) consists of a five-stranded anti-parallel β-sheet, while in α-HCoVs, it forms β-sandwich with six strands.

### Alpha-Coronaviruses

HCoV-229E and HCoV-NL63 from α-HCoV were first discovered in 1966 and 2004, respectively (Chiu et al., 2005). HCoV-229E was originated in bats and transferred to camelids and alpacas (Corman et al., 2015), while HCoV-NL63 was originated in bats (Donaldson et al., 2010; Huynh et al., 2012). In HCoV-229E, over the past fifty years due to natural variation of receptor-binding motif, there appeared six RBD classes (class I-VI) in the entire viral genome, which successively evolved and replaced each other (Wong et al., 2017). The overall structure of the 229E and NL63 spike presents a similar trimer by β-sandwich fold (Figure 2A). The homotrimeric S1 subunits form a triangular cap over the trimeric S2 subunits, and each S1 connects to the S2 of an adjacent monomer by a non-covalent bond, in which domain A sits at the vertices and domain B (RBD) is located close to the axis of the triangle cap (Li et al., 2019).

Unlike β-coronaviruses and 229E, NL63 of α-HCoV presents an additional canonical domain 0 adopting β-sandwich fold with a three-stranded β-sheet (Walls et al., 2016), which may be the result of gene duplication. The NL63 spike ectodomain shows a packed NTD and CTD organization (Yuan et al., 2017). The RBD of NL-63 presents a unique β-sandwich core structure consisting of 2 layers β-sheets with each three stranded, stacked against each other by an extensive hydrophobic bond, presenting 3 discontinuous receptor-binding motifs (RBM1: β1-β2, RBM2: β3-β4 and RBM3: β5-β6) to bind to the human angiotensin converting enzyme 2 (ACE2) receptor (Wu et al., 2009). The N-terminal two lobes of hACE2 form a claw-like structure showing open and closed conformation (Towler et al., 2004). All 3 discontinuous RBMs forming 3 β-loops responsible for receptor binding are connected to short β-strands surrounding a shallow bowl-shaped cavity (Wu et al., 2009), which is opposite to the receptor-binding interface and can stabilize the distal end of the RBD. Among them, the RBM1 (β1-β2) of NL63-CoV RBD with a stable disulfide bridge makes extensive interactions with hACE2 (Guruprasad 2021a), while other coronaviruses RBM1 in group I loses a critical disulfide bond, but RBM2 adds cysteines and is likely to form a disulfide bond, which may help to bind to human aminopeptidase N (APN) rather than ACE2.

### Beta-Coronaviruses

Human OC43 originates from a host-range variant of *Beta-*coronavirus. HCoV-OC43 and HCoV-HKU1 from lineage A of β-hCoV were first discovered in 1967 and 2005 respectively (Vlasak et al., 1988; Woo et al., 2005), and they were originated in rodents (Su et al., 2016; Forni et al., 2017). Both hCoVs can bind to 9-*O*-acetyl-sialoglycan by domain A of spike (Hulswit et al., 2019). The cryo-EM structure of the HCoV-OC43 spike shows that the S1 subunit presents a V-shaped architecture by the A, B, C, and D domains (Tortorici et al., 2019). The five-stranded anti-parallel β-sheet core and highly variable external loop of domain B mediates receptor binding in MERS-CoV, SARS-CoV-1, and SARS-CoV-2 spikes (Hatmal et al., 2020), whereas domain A of HCoV-OC43 presents an alternative β-sandwich architecture (Tortorici et al., 2019). The spike trimer structure of HCoV-HKU1 reveals a woven NTD-CTD swapping organization. HKU1 S1 NTD subunit (domain A) can bind to *O*-acetylated sialic acids on host cells by an extended conformation with short linkers, which are critical receptor determinants for the hCoV-HKU1 infection (Huang et al., 2015). However, with canonical architecture consisting of a structurally conserved core and a variable loop, HKU1 S1 CTD (domain B) is buried in the spike trimer and covers S2 central helix in pre-fusion state. This conformation lacks equivalent interactions and prevents the fusion action (Kirchdoerfer et al., 2016). In fact, domain B in HCoV-OC43, HCoV-NL63, and HCoV-HKU1 present only closed conformation (Tortorici et al., 2019).

MERS-CoV of lineage C of HCoV was identified in 2012 (Zaki et al., 2012), and it was considered to originate from bats and transmitted from dromedary camels to humans (Alagaili et al., 2014; Azhar et al., 2014; Hu et al., 2015; Cui et al., 2019). The cryo-EM structure of MERS and its complex with receptors was determined. The S1 domain A of MERS-CoV is involved in sialic acid binding to activate hemagglutination (Li et al., 2017), causing human erythrocyte agglutination. The receptor-binding subdomain of S1 domain B comprising of four stranded anti-parallel β-sheet (β5- β8), are responsible for binding to human dipeptidyl peptidase 4(DPP4) receptor, which is a transmembrane serine protease expressed in human airways epithelial cells, with N-terminal eight-blades β-propeller (Gallagher and Perlman 2013). The long loop between β6 and β7 of MERS-CoV domain B is perpendicular to the β sheet and provides structural support to contact DPP4 (Wang et al., 2013). The cryo-EM structure of MERS-CoV reveals two different conformations of the spike trimer with one or two of the three S1 RBDs in the “standing” state, and the other parts remain the same (Yuan et al., 2017). However, the disassociated S1 trimer forms a ring like structure with NTD, flexible RBD, and subdomains 1 and 2 with three standing RBD domains, among which the NTD provides a stable triangular platform for the flexible RBD located on the triangular edges. Thus, the S1 trimer with three standing RBD domains is easy to dissociate from the S2 subunit, which is responsible for receptor binding (Yuan et al., 2017).

SARS-CoV-1 and SARS-CoV-2 from lineage B of β-HCoV were erupt in 2002 (Drosten et al., 2003; Zhong et al., 2003) and in 2019 (Huang et al., 2020; Zhou et al., 2020). SARS-CoV-1 was transmitted by bats and palm civets (Song et al., 2005; Wang et al., 2005; Hu et al., 2017). Similar to other HCoV structure folds, the SARS-CoV-1 S form trimer, although SARS-CoV-1 spike remains uncleaved due to a lack of a furin cleavage site (Rota et al., 2003; Li F. et al., 2005). SARS-CoV-1 RBD comprises a core consisting of five stranded anti-parallel β-sheets stabilized by 3 short -helices; and a receptor-binding motif (RBM) consisting of extended loop to form two ridges and two-stranded anti-parallel β-sheet (Li F. et al., 2005). The structure of the SARS-CoV-1 spike trimer reveals two classes: one is all three S1 RBDs in the lying state (close); the other is two lying RBDs and one standing RBD (open). In spike trimers of MERS-CoV or SARS-CoV-1, the RBD in the open state features weaker and poorer density, indicating flexibility for receptor binding (Yuan et al., 2017). In the standing state, the receptor binding surface is exposed to bind to the receptor. The RBM of spike features a concave surface to cradles the ACE2 helix and mediate binding to ACE2 by a short, two-stranded antiparallel β-sheet and two loop ridges (Li F. et al., 2005). The structure of the SARS-CoV-1 RBD complex with hACE2 further demonstrates that the interaction of the standing RBD with the receptor facilitates the dissociation of the S1 subunits (Song et al., 2018).

SARS-CoV-2 originated from bats and pangolins should be considered as possible hosts (Lam et al., 2020; Zhou et al., 2020). The S proteins and even the whole genome of SARS-CoV-2 and SARS-CoV-1, which are phylogenetically closely related and structurally conserved, have an amino acid sequence identity of 77 and 79.6%, respectively (Hoffmann et al., 2020; Zhou et al., 2021), while it reduces to 50% the identity of their RBMs (Hatmal et al., 2020). The structural analysis of the SARS-CoV-2 full-length spike protein: ACE2 complex shows two spike trimers simultaneously bind to an ACE2 homodimer (Yan et al., 2020). SARS-CoV-2 S1 RBD undergoes a hinge-like movement to transition between “up” and “down” conformations (Wrapp et al., 2020). Spike ectodomain of SARS-CoV-2 shows a 160 Å long trimer with a triangular cross-section (Wang Q. et al., 2020). The overall structure of SARS-CoV-2 resembles that of SARS-CoV-1, but there are minor differences in RBD position in down conformation. In down conformation, SARS-CoV-1 RBD is tightly bound to the NTD of the neighboring protomer, while SARS-CoV-2 RBD presents an angle closer to the central cavity of the trimer (Wrapp et al., 2020), which may affect the receptor affinity. Similar to SARS-CoV-1 five-stranded antiparallel β-sheets core domain, the two short β5 and β6-strands connected by α4 and α5 helices appear in SARS-CoV-2 RBD (Lan et al., 2020) while the RBM of SARS-CoV-1 is lacking of β6 strand and β7 strand. The external loop covering the most contacting interface of SARS-CoV-2 with ACE2 is highly variable (Lan et al., 2020).

## The Predominant Receptor of Human Coronavirus Spikes

Cornaviruses enter into the host by a mechanism of receptor recognition. These human coronaviruses recognize different receptors through the spike S1 RBD subunit. Human coronaviruses from the same or different genera can utilize different and the same receptors by conservative or independent receptor recognition mechanism (Li et al., 2017). As shown in Table 1, HCoV-229E and HCoV-NL63 from *Alpha-coronavirus* are related but recognize different human APN (Yeager et al., 1992; Bonavia et al., 2003) and ACE2 receptors (Li et al., 2003; Wang Q. et al., 2020), respectively. The NL63 coronavirus is the only one to bind to ACE2 in group I coronaviruses. Other Group I coronaviruses all use APN as a receptor, which is from their respective host. Meanwhile, NL63 also binds to heparan sulfate proteoglycans to participate in virus anchoring by domain 0 or domain A (Milewska et al., 2014). In lineage B of *Beta-coronavirus*, both SARS-CoV-1 and SARS-CoV-2 recognize ACE2 (Hofmann et al., 2006; Wang Q. et al., 2020), but MERS-CoV from lineage C recognizes DPP4 by domain B (Raj et al., 2013) and binds to sialic acid by domain A. HCoV-OC43 and HCoV-HKU1 from lineage A of *Beta-*coronaviruses specially bind to 9-*O*-acetyl-sialic acid (9-*O*-Ac-Sia) (Schultze et al., 1991; Hulswit et al., 2019).Therefore, it is necessary to further explore the mechanism of interaction between coronavirus and receptor to elucidate human coronavirus pathogenesis and cross-species potential.

The core and highly variable loops of domain B mediate protein receptor binding in MERS, SARS-CoV-1, SARS-CoV-2, 229E, and NL63. The peripheral groove of domain A is involved in interaction with the ligand sialic acid in OC43, HKU1, and MERS. The understanding of the receptor recognition mechanism facilitates elucidation of viral infectivity and pathogenesis, which is a major target of designing vaccines and antiviral drugs.

## Structural Mechanism of ACE2 Receptor Recognition by Spike-RBD in Human Coronaviruses

HCoV-NL63 uses heparan sulfate proteoglycans to attach to target cells and participate in virus anchoring and infection (Milewska et al., 2014; Walls et al., 2016). Domain 0 or A of NL63 spike is responsible for binding to heparan sulfate, which might activate HCoV-NL63 spike and further promote interactions with the ACE2 receptor (Milewska et al., 2014). Surprisingly, the S1 RBD (core and RBM) from *alphacoronavirus* and *betacoronavirus* have no obvious sequence and structural homology, but the S1 RBD of both HCoV-NL-63 and SARS-CoV-1 can share their common ACE2 receptor with high affinity (Kd of 34.9 and 31 nM, respectively), although this binding interface of NL63-CoV is slightly smaller than that of SARS-CoV-1 (Wu et al., 2009). This may be another independent way to recognize common receptors. In fact, the spike RBD core is conserved but the RBM is variable in group I coronaviruses. In the core β-sandwich structure, 2 disulfide bonds by 4 cysteines are formed to strengthen conformation. HCoV-NL63 RBMs comprise 3 short, discontinuous β-loops connecting to β-strands and surrounding a shallow bowl-shaped cavity (Wu et al., 2009), while SARS-CoV-1 RBM comprises a long, continuous loop located at one edge of the core (Figure 2C) (Li F. et al., 2005). Correspondingly, 3 discontinuous virus-binding motifs on ACE2 are defined as VBMs. NL63-CoV RBMs show more extensive interaction with VBM2 and VBM3 of the hACE2 receptor, but less contact with VBM1. The complex structure of NL63 RBD with ACE2 shows that the VBM3 is inserted into the bowl-shaped cavity of RBD (Wu et al., 2009). The interaction is directly mediated by 11 viral residues and 16 receptor residues, with a slightly smaller binding interface but similar binding affinity compared with SARS-CoV-1. Despite the different structures of spike CTD between HCoV-NL63 and SARS-CoV-1, they recognize the same 3 VBMs on ACE2, although the latter can recognize one more VBM1b (Figure 2B). In addition, there is a virus-binding hotspot on ACE2 involving Lys353, which is the key to the binding of NL63 and SARS-CoV-1. Upon NL63 binding, the Lys353 residue is embedded in a hydrophobic tunnel formed by NL63 Tyr498 and Ser535 (Figure 2B). This hydrophobic interface is conductive to the salt bridge formation by Lys353 and Asp38 on ACE2. The substitution or mutant involving hotspot structure changes can abolish NL-63-CoV binding. This hotspot site of ACE2 is also critical for the SARS-CoV-1’s affinity to host receptor and pathogenesis, although it happens with the mutation of Ser535 and Tyr498 to Thr487 and Tyr491 on SARS-CoV-1, respectively (Wu et al., 2009) that have the same properties and interaction. This is a strikingly similar structural mechanism of receptor recognition between 2 different viruses. These hotspot sites are highly conserved and invariable, which provides clues for drug development. Compared with spike RBD of SARS-CoV-1 isolated during severe 2002–2003, it present ∼10-fold lower affinity with Kd of 352 nM of ACE2 receptor with S RBD SARS-CoV-1 isolated during mild 2003–2004 by mutation of T487S (Li W. et al., 2005; Wu et al., 2009). Thus virus-binding hotspots on the receptor and the receptor-binding hotspot on the virus are the binding targets of the virus, which determine the viral pathogenesis and infection, and thus become the targets of drug design.

There are 16 residues in SARS-CoV-1 RBD that participate in interaction with hACE2. The key determination factor of species transmission depends on the interaction of SARS-CoV-1 RBD involving Asn479 and Thr487 with ACE2 involving Lys31, Asp38, Tyr41, and Lys353 (Figure 2B) (Li F. et al., 2005; Wu et al., 2011). SARS-CoV-1 does not infect or infect inefficiently other animals with ACE2 that possessing different residues, such as mouse (Li et al., 2004). The structural basis of SARS-CoV-1 interaction with ACE2 provides novel clues for cross-species transmission outbreaks and coronavirus epidemic outbreaks. The mutation of N479K and T487S damages viral affinity for the hACE2 receptor and decreases viral infectivity in human beings (Li W. et al., 2005; Qu et al., 2005). This explains the mild hGd03 SARS infection in 2003–2004, compared to the severe hTor02 infection in 2002–2003. Therefore, the hotspots on virus RBD and receptors that participate in their interaction determine viral infectivity, transmissibility, and pathogenesis. In addition, the spike trimer of SARS-CoV-1 connects the tip of the ACE2 lobe rather than occluding the peptidase active site. Thus the specific ACE2 inhibitors targeting its activity site cannot affect the interaction of spike with ACE2 (Li W. et al., 2005).

The spike of SARS-CoV-2 and SARS-CoV-1 share the same functional host cell receptor, ACE2. The overall conformational structure and the binding mode of RBD to ACE2 are also nearly identical (Figure 2C). SARS-CoV-2 spike trimer has ∼10- to 20-fold higher affinity with the dissociation constant (Kd)∼15 nM to ACE2 receptor than that of SARS-CoV-1 with Kd of 325.8 nM (Wrapp et al., 2020), which may contribute to its increased virulence. Compared with SARS-CoV-1, 17 residues in the recent SARS-CoV-2 RBD participate in interaction with hACE2. Out of 16 residues that bind to ACE2 in SARS-CoV-1, 8 amino acid residues are strictly conserved in SARS-CoV-2 (Figure 1C) (Li et al., 2003; Yan et al., 2020). SARS-CoV-2 RBM forms a gently concave surface with a ridge on one side, which is complementary and in contact with the exposed outer surface of the claw-like hACE2 (Shang et al., 2020). Most RBMs are located in α4, α5-helices, β5, β6-sheets, and the connecting loops of SARS-CoV-2 RBD (Lan et al., 2020; Shang et al., 2020). SARS-CoV-2 RBD forms a broader binding interface and more atomic interactions with hACE2, which indicates a more favorable interaction with hACE2 with the low dissociation constant (kd) (Lan et al., 2020). By comparative analysis of known structure complexes, it showed that SARS-CoV-2 RBM (as Figure 2A, terminal region 1, middle region, and terminal region 2) presents more sequence variation and an obvious structure change compared with that of SARS-CoV-1 (Hatmal et al., 2020). The RBM of SARS-CoV-1 lack of β6 strand and β7 strand in terminal region 1, while in the SARS-CoV-2, followed β6, there is an Ala475 residue that is involved in hydrophobic interaction with β7 Tyr489 and ACE2 (Figure 2B). It is proline residue (Figure 2C) at the same site as the SARS-CoV-1 RBD that leads to loop formation.

## Mutation Hotspot of SARS-CoV-2 RBM

Genotyping analysis of RBM from different human coronaviruses and SARS-CoV-2 RBM isolated around the world revealed that RBM-relative genes undergo frequent mutations in highly variable region, which determine virus-affinity to host receptor and infectivity (Yin 2020). But some residues are relatively conserved in receptor-binding interfaces, which provide cues for vaccine development and therapeutic drug development. Neutralizing antibodies can bind to antigens of pathogens’ surfaces and prevent them from adhering to host cell receptors, thus inhibiting infection. The neutralizing antibodies that tolerate broadly RBD mutations provide a potential against pathogens including SARS-CoV-2.

The most prominent mutation in the middle region is the substitution of Val404 of SARS-CoV-1 RBD with Lys417 in the SARS-CoV-2 RBD, which may result in a higher affinity of RBD with a Kd of 4.7 nM to the receptor by salt bridge formation of Lys417 with Asp30 of ACE2. The Kd between SARS-CoV RBD and ACE2 is 31 nM (Lan et al., 2020). Thus, Lys417 residue with a positive charge is critical to stable core conformation and enhances the binding affinity to the ACE2 receptor. It is reported that this Lys417 mutation hinders the neutralizing activity of the SARS-CoV-1 antibody to SARS-CoV-2 (Yan et al., 2020). Some other mutations in the middle region, such as Ile489/Val 503 and Asp393/Glu408, have less effect since they have the same properties and interaction contacts. However, the Arg426 on SARS-CoV-1 mutation to Asn439 on SARS-CoV-2 eliminates salt bridge formation, resulting in weak interaction of RBD with Asp329 of ACE2. Additionally, terminal region 1 of SARS-CoV-2 is critical for binding, especially Cys480, Val483, Phe486 and Cys488 active residues that can be targeted for druggability and vaccinability. The mutation of Leu472 of SARS-CoV-1 to Phe486 of SARS-CoV-2 can weaken van der Waals interactions with Met82 of ACE2 (Yan et al., 2020). Thus the electrostatic interaction is stronger in SARS-CoV-2-ACE2 complex than that in SARS-CoV-1, making it greater binding affinity (Hatmal et al., 2020). In the middle shallow pit of SARS-CoV-2 RBM, it provides binding interfaces for small molecular drugs, for example, hesperidin, which can target the central shallow pit on spike-ACE2 binding interface (Hatmal et al., 2020). In addition, the other mutations in this domain, including Ser494 and Leu452, increase the hydrophobicity of the shallow pit (Figure 2B). The other terminal regions (TR2 and GFQPTNGVG in SARS-CoV-2; GFYTTTGIG in SARS-CoV-1) also involve critical interactions with ACE2. Among which, the mutation to proline residue (P499) on SARS-CoV-2 that forms GXXP and PXXG motifs can form a sharp kink to affect RBM–ACE2 interaction. Tyr505 in SARS-CoV-2 or Tyr491 in SARS-CoV-1 RBM is conserved amino acid (Hatmal et al., 2020).

In the process of a pandemic, adaptive mutations in the SARS-CoV-2 could confer infectivity and alter its pathogenicity (Korber et al., 2020; Zhang et al., 2020), which increases the difficulty of drug development but attaches great consideration. The highest frequency of mutation occurs near the RBD-ACE2 interface. From the *Delta* to *Omicron*, it presents highly conserved and important point substitutions (D614G, E484K (G142D), K417N, and N501Y), which influence hACE2 binding affinity and human transmissibility but decrease severity and efficacy to vaccine and antibody (Luan et al., 2021; Wang et al., 2021; Papanikolaou et al., 2022). The D614G mutation in S1 domain D is the most prevalent and occurs at a high frequency. The change of D614 locating on the surface of the spike to G614 eliminates side-chain hydrogen bonds and increases the number of RBDs in the up conformation, which results in increased RBD exposure and encounter with the ACE2 receptor (Mansbach et al., 2020). The RBD of the SARS-CoV-2 spike presents 44 distinct mutation sites (Guruprasad 2021b). Mutation residues at Y453, G476, F486, T500, and N501 that are located close to the ACE2 receptor interaction interface would affect surrounding protein charge and the structural conformation, which is important for vaccine design by spike protein epitope exposure (Guruprasad 2021b). The cryo-EM structure of the Omicron variant spike–ACE2 complex revealed that the overall conformation of the trimer is similar to the wide type strain, and mutations are mainly distributed on one face of RBD, which spans the ACE2-binding region and epitopes being targeted for neutralizing antibodies (Mannar et al., 2022).

**FIGURE 3:**
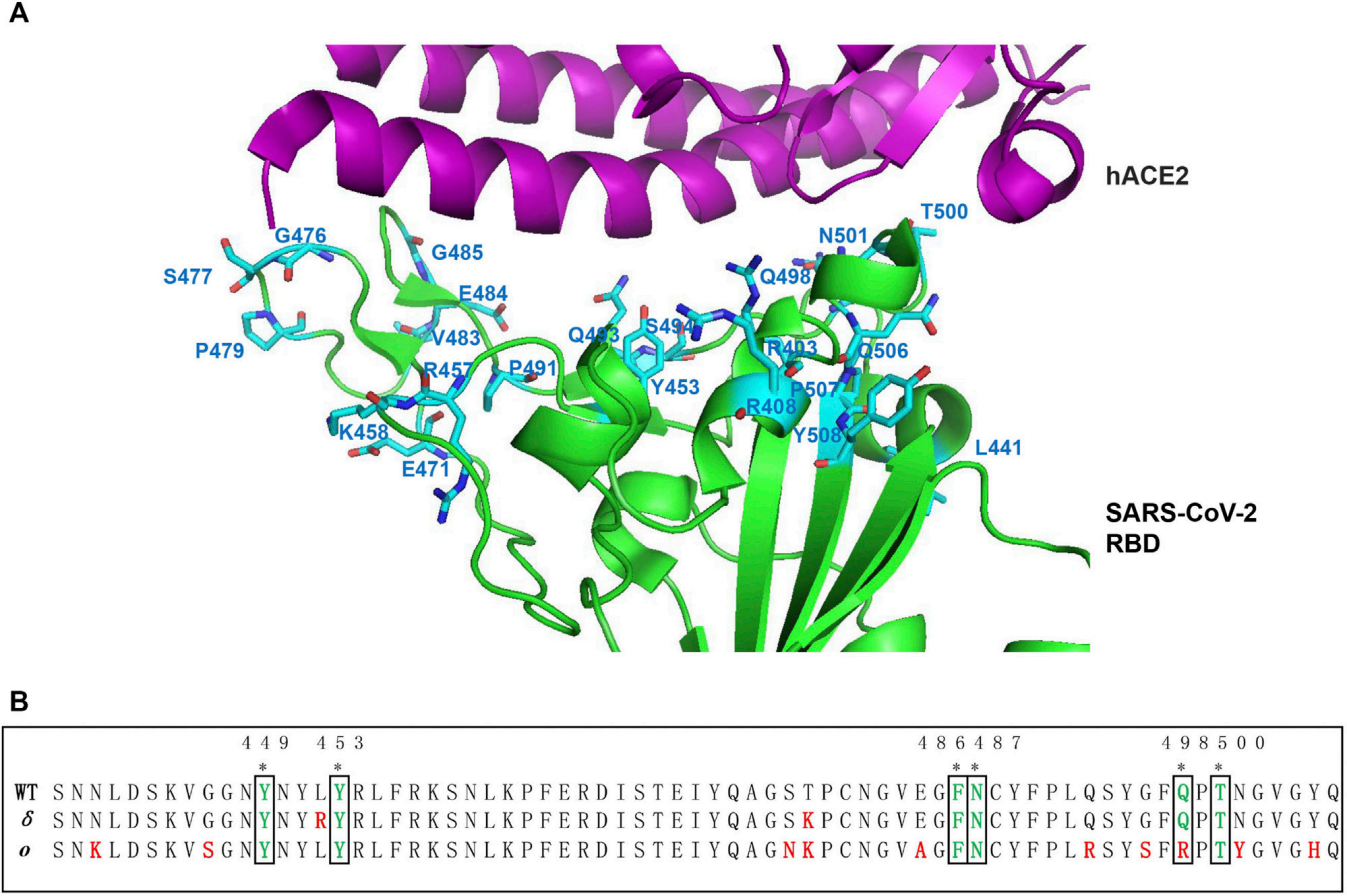
Mutation hotspot of SARS-CoV-2 RBM. **(A)** Compared with SARS-CoV-2 from Wuhan-HuBei-1, the mutation hotspot on ACE2 interaction sites of SARS-CoV-2 RBM is mapped on to the crystal structure of RBD complex with ACE2 side-chain residues (PDB code: 6LZG) (Wang Q. et al., 2020). The 22 suggested ACE2 interaction mutation residues including R403, R408, L441, Y453, R457, K458, E471, G476, *S477*, P479, *V483*, E484, G485, P491, Q493, S494, Q498, T500, *N501*, Q506, P507, and Y508 are shown in blue (Guruprasad 2021b). Among which, S477N, V483A, and N501Y are more frequent mutations. **(B)** Sequence alignment of ACE2 interaction residues on RBM between WT, Delta, and Omicron variant. The 6 common ACE2 interaction sites were indicated by asterisk on the top. The mutation residues in the RBM of *Delta* and *Omicron* variants were indicated by red color.

There are 22 suggested ACE2-interacting residues in the receptor-binding motif (Figure 3A). Compared with WT, the six common ACE2 interaction sites were conserved and invariant in SARS-CoV-2 RBM variants (Alpha-Kappa), including Y449, Y453, F486, N487, Q498, and T500 (Figure 3B). However, two common mutation sites (E484K/Q and N501Y) are present in ACE2 interaction sites in ten SARS-CoV-2 variants RBM (Jhun et al., 2021). In the Alpha variant of SARS-CoV-2, it reports 3 mutation residues in RBD, including E484K, S494P, and N501Y, while in the Beta variant, it adds a K417N (Jhun et al., 2021; Papanikolaou et al., 2022). But K417 is substituted by T but not N in the Gamma variant RBD. There are three common mutations (L452R, D614G, and P681H/R) shared by Delta, Kappa, and B.1.617.3 variant of SARS-CoV-2. The common L452R mutation occurs in Delta, ETA, IOTA, Kappa, and B.1.617.3 variants. The E484Q is found in Kappa and B.1.617.3 variants. The unique mutation site for Delta variant: T478K within the SARS-CoV-2 RBM occurred, but its association with the recent spread worldwide outbreak still needs to be explored. Therefore, hotspot mutation in the RBM may highly influence the infectivity and pathogenicity of recent SARS-CoV-2, which become the focus of designing drugs and vaccines against virus (Jhun et al., 2021; Papanikolaou et al., 2022). Recent studies reported that the Delta variant likely confers resistance to available vaccines or monoclonal antibodies (Weisblum et al., 2020; Yadav et al., 2021), and that individuals previously infected were probably more susceptible to being reinfected by the Delta variant. Vaccines based on the SARS-CoV-2 Alpha variant may provide the broadest protection, although the correlation with the mutation site is uncertain (Liu et al., 2021). The new mutations at Q493R, G496S, Q498R, and N501Y appear to form new salt bridges and hydrogen bonds to restore ACE2 binding affinity that decreased in the K417N variant with a Kd of 75nM, while it can escape antibody neutralization. In addition, more mutations occurring in the RBM/ACE2 interaction interface in the recently dominant and fastest transmissible Omicron variant of SARS-CoV-2, including N440K, G446S, S477N, T478K, E484A, Q493R, G496S, Q498R, N501Y, and Y505H, compared with the Delta variant (Figure 3B), may eventually result in high transmissibility of the variant (Kim et al., 2021).

## Structural Basis for HCoV-OC43 and HCoV-HKU1 Attachment to Sialic Acid Receptors

HCoV-OC43 and HCoV-HKU1 bind to 9-O-acetyl-sialic acid (9-O-Ac-Sia) receptor by S1 domain A, presenting a β-sandwich architecture, which links to oligosaccharides located on the cell surface (Huang et al., 2015). Upon binding to the viral, 9-O-Ac-Sia buries a small surface area (300–400Å^2^) into the spike groove (Tortorici et al., 2019). The HCoV-OC43 spike interacts with the 9-*O*-Ac-Sia producing two hydrophobic pockets, which are delineated by two loops forming the rims of the binding site (loop1: 27-Asn-asp-Lys-Asp-Thr-Gly-32, and loop2: 80-Leu-Lys-Gly-Ser-Var-Leu-Leu-86) in a periphery groove of domain A (Figure 2B). This ligand-binding groove is mediated specifically by the interaction of the carbonyl moiety of 9-*O*-Ac-Sia with the side chains of domain A through hydrogen bonding and salt bridge (Tortorici et al., 2019).

The sequence identity of Domain A between OC43 and HCoV-HKU1 S1 is only 55–60%, but they use the same binding sites to interact with 9-O-Ac-sialoglycans (Hulswit et al., 2019). The corresponding binding sites on the 9-O-Ac-Sia receptor are also conserved and functional (Tortorici et al., 2019). However, the binding sites of HKU1 domain A residing at the bottom of the canyon contribute to significantly lower binding affinity measured by high-sensitivity nanoparticle-hemagglutination assay than that of OC43 (Hulswit et al., 2019). Thus structure basis with the conserved binding pocket and identical interaction to ligand applies to all coronaviruses with 9-*O*-Ac-Sia as a receptor, but still show different characteristic due to local architecture. In addition, it was reported that domain B in HCoV-OC43, HcoV-NL63, and HCoV-HKU1 presents only a closed conformation, which is blocked from binding to the receptor (Tortorici et al., 2019). Thus, further exploration is needed to reveal the interactions of these OC43 and HKU1 HCoVs domain B with unknown human protein receptors during the entry process and infection mechanism. There must be important factors to activate conformational changes to expose receptor-binding sites to bind unknown human receptors during dynamic virus progress.

Other coronaviruses like MERS-CoV also bind to sialoglycans (They are non-*O*-acetylated-sialoglycans) *via* domain A, but the ligand-binding pocket is not conserved with that of HCoV-OC43 (Li et al., 2017), and it involves different interactions with non-acetylated-sialoglycans. MERS-CoV S1 domain A has a binding preference for α 2, 3-coupled, 5-N-acetylated neuraminic acid, suggesting that it may involve different interactions in the same domain along with their evolution and adaptation, and this adjustment may affect transmission and infection of coronavirus.

## Structural Mechanism of Receptor Recognition of Other Human Coronaviruses

Except for binding to sialic acid by S1 domain A, MERS-CoV can efficiently infect bats (Lau et al., 2013), camels (Alagaili et al., 2014; Barlan et al., 2014; Haagmans et al., 2014) and humans by binding to DPP4 receptors through S1 domain B. The complex structure of MERS-CoV with DPP4 has also been determined (Wang et al., 2013). Different from the core conformation introduced previously, RBD of MERS-CoV forms four-stranded anti-parallel β-sheet core conformation presenting a flat surface which mediates binding to DPP4. The blade 4 and blade 5 of DPP4, which contains eight-bladed β-propeller domain specifically bind to RBD of MERS-CoV with two patches (Figure 2B). The patch 1 is formed by the interaction of the C-terminal long loop of β6 - β7 with the blade 4. In patch 2, a concave outer surface is formed byβ5- β8 strands and the β5-β6 loop. K267, R336 and R317 residues with positively charge on the outer surface of blade 4 and blade 5 of DPP4, but not other blades, can interact with D510, E536, D537, and D539 residues with negatively charge on the surface of RBD, with the short α helix between blade 4 and blade 5 docking into the hydrophobic concave of RBD. The sequence variation of VBM residues of DPP4 from different species may help to determine the host range of MERS-CoV (Wang et al., 2013). The sequence identity of RBD between SARS-CoV-1 and MERS-CoV is poor with 24%, although conservation in the core structure, which indicates different host cell receptor specificity, which is critical for cell tropism and pathogenesis (Wang et al., 2013). Correspondingly, targeting the binding of RBD and receptors, there are several strategies to restrain MERS-CoV pathogenesis.

The crystal structure of the HCoV-229E RBD (Class I, III, IV, and V) in complex with hAPN reveals three extended loops on RBD are responsible for binding to the hAPN receptor (Wong et al., 2017; Li et al., 2019). However, loop1 mediated conserved core interaction to bind specially to hAPN (Bonavia et al., 2003). The 287-291 residues on hAPN presenting a surface exposed β-strand interact with S312-C320, R359, W404, S407, and K408 residues of RBD (Figure 2B). The sequence variation of RBD classes concentrated in the peripheral region that form loop plasticity but the other preserved the core interactions. Thus HCoV-229E RBD classes show different affinity to APN receptor with an ∼16-fold range Kd from ∼30 to ∼440 nM or neutralizing antibody. The affinity of Class I RBD to hAPN is lowest, while it is highest in the Class V and VI RBDs (Wong et al., 2017; Li et al., 2019). Interesting, the hydrogen bonds formation during the interaction RBD with hAPN is involving in backbone atoms of hAPN, which leads to independence on a given sequence, and increases the chances to bind to homologous receptor. In the cryo-EM structure of 229E RBD, domain B in all three monomers of the trimer is in the down conformation, with a blocked receptor binding end, which may not be possible for receptor binding. Nevertheless, it is likely mediated by the up/down conformational conversion in 229E, with gain and loss of interaction between domain B and C, as well as domain B and S2. Both of these two interactions are involved in stabilizing RBD in the down conformation (Li et al., 2019).

## Conclusion and Therapeutic Strategies by Targeting Spike RBD Protein

During the past five decades, 229E, NL63, and OC43 of the human coronaviruses caused mild and self-limiting infections, while SARS, MERS, and SARS-CoV-2 caused severe respiratory tract infection, even mortality. The spike protein covering the virus surface offers a good druggable target to impede binding to the host cell and subsequent internalization and infection. Thus, the understanding of the structure and mechanism can provide more effective therapeutic strategies for combating these infectious diseases by targeting spike RBD. The S1 domain A of HCoV-HKU1 and HcoV-OC43 spike are responsible for binding sialic acid to mediate CoV–host interaction, while the protein receptors have not been identified yet. S1 domain B of 229E, NL63, SARS-CoV-1, and SARS-CoV-2 are involved in specifically recognizing different cell protein receptors. MERS can bind to sialic acid and DPP4 by domain A and domain B, respectively.

Human CoV genomes undergo various mutations and recombination during evolution, including spikes (Menachery et al., 2016), to facilitate transmission, infect, and adapt to human hosts. In this review, we have presented structural and mutational characteristics of the human coronavirus spike RBD and their interaction with receptors. Understanding the mutation rules and conservatism in RBM interaction with the receptor provides critical cues for developing antiviral drugs. In general, the S protein is a homotrimeric glycoprotein in a pre-fusion and post-fusion conformation. When S1 binds to a host cellular membrane receptor, the spike undergoes a substantial structural rearrangement and dissociates from the S2 subunit, which transits to a stable post-fusion conformation. However, in this process, it involves different domains and different interactions among human coronaviruses, which may explain their different tendencies for binding receptors and infecting hosts. Meanwhile, compared with SARS-CoV-1, 17 residues in the recent SARS-CoV-2 RBD participate in interaction with hACE2, among which 8 residues are completely consistent and highly conserved. Other mutated residues, including V405/K417, affect receptor-binding affinity and even infectivity. In defined variants of recent worldwide SARS-CoV-2 (Alpha to Omicron), RBD mutation became a concern for vaccine development. In fact, more unique mutations occurred in the global epidemic omicron variant, which shows stronger infectivity. However, whether it is caused by mutation needs to be verified. The continuous mutation of coronavirus brings many challenges to current antibody drugs.

Many studies report that antibodies targeting RBD produce effective neutralizing responses to treat viral infection. In 2020 and 2021, a total of five monoclonal antibodies were approved or authorized for emergency application in the treatment of COVID-19. Bamlanivimab (LY-CoV555) is the first approved monoclonal antibody that binds to RBD for the treatment of mild to moderate COVID-19 (Chen et al., 2021). However, its curative effect is not obvious for severe and critical COVID-19 patients. The receptor-binding motif (RBM) that specifically interacts with human receptors can be used as a candidate epitope for antibody design. Currently, many vaccine candidates based on spike RBD are entering clinical trial, including recombinant protein, DNA or mRNA vaccines (Amanat and Krammer, 2020; Wang F. et al., 2020; Yu et al., 2020), pointing out the important role of RBD for vaccination against SARS-CoV-2. Vaccinated individuals by the RBD vaccine could generate antibodies targeted to the pre-fusion conformation and hinder binding to ACE2 and further access to cells, which reduces nonspecific antibody production. More than 40 nanobodies against SARS-CoV-2 targeting to RBD interaction with ACE2 were under investigated, which can recognize epitopes that are usually unavailable to traditional antibodies and avoid virus immune escape. A study reported that the humanized monoclonal antibody targeting S RBD conserved epitopes, H014, prevents attachment of SARS-CoV-2 to its host cellular receptors in a mouse model (Lv et al., 2020), and could be used as a as therapeutic antibody in the treatment of COVID-19. This designed antibody targeting on conserved epitopes of RBD may be effective to cross-neutralize other lineage B coronavirus. STE90-C11 antibody derived from human IgG1 with FcγR-silenced Fc can tolerate most known emerging RBD mutations, and inhibit SARS-CoV-2 binding to ACE2 (Bertoglio et al., 2021), which could treat severe COVID-19. Correspondently, another therapeutic strategy targeting the host receptors with antibodies or inhibitors also provides possibility to block receptor engagement. David’ lab designed a multivalent ACE2-mimic AHB2 (TRI2) protein inhibitor, which can broadly neutralize Omicron, Delta and all other variants (Hunt et al., 2022). This structure-guided inhibitor design is rapid and optimal with great prospects, although it needs to go through long-term clinical trials to assess its effectiveness and safety. In addition, small-molecule inhibitors and antibodies can be designed to target the amino acid residues involved in protein interactions between hCoV spike and receptors. Shi et al. reported a specific human monoclonal antibody CB6 that interferes with SARS-CoV-2 RBD-ACE2 interaction by recognizing an epitope that overlaps with ACE2-binding sites in RBD (Shi et al., 2020). These strategies show promise only for a specific target, including mutant variants. For human coronaviruses from different genera, NL63 and SARS-CoV recognize the same receptors by an independent mechanism. This gained experience of therapeutic interventions based on RBD structural information for SARS-CoV-2 is likely to facilitate the development of antibodies and inhibitors against various other coronaviruses.

Furthermore, the amino acid sequences connecting S1 and S2 subunits in the hCoV spike are variable, and the ‘PRRA’ furin cleavage motif of SARS-CoV-2 plays critical roles in enhancing infectivity and COVID-19 pathogenesis (Coutard et al., 2020; Hatmal et al., 2020; Johnson et al., 2021). MERS-CoV spike contains the ‘RSVRSV’ cleaved motif (Millet and Whittaker 2014), while SARS-CoV-1 spike lacks of favorable cleavage site and is uncleaved (Li F. et al., 2005). In addition, another furin-like cleavage site on S2 (S2′ cleavage site) appears in SARS-CoV-2, SARS-CoV-1, and MERS-CoV (Hatmal et al., 2020). Thus, targeting furin enzyme and cleavage site may interfere virus processing of entering into host cells including HKU1-CoV, OC43-CoV, MERS-CoV, and SARS-CoV-2 (Johnson et al., 2021). A study reported that Furin inhibitors, decanoyl-RVKR-chloromethylketone (CMK), block SARS-CoV-2 entry and suppress cleavage of spike but do not disrupt the binding of SARS-CoV-2 to ACE2 (Cheng et al., 2020). Loss of furin substantially reduces S1-S2 cleavage but it does not prevent it, which indicates furin inhibitors may reduce but not abolish viral spread (Papa et al., 2021). If the mutation of the furin site occurred in dominant epitopes, it may reduce the interaction of spike and ACE2 and alter the targets for antibody generation (Wrobel et al., 2020). Alternative prevention and therapeutic strategies against human coronaviruses, including SARS-CoV-2, continue to be an urgent need for solving the present and future HCoV outbreaks.
